# Trends of notification rates and treatment outcomes of tuberculosis cases with and without HIV co-infection in eight rural districts of Uganda (2015 – 2019)

**DOI:** 10.1186/s12889-022-13111-1

**Published:** 2022-04-05

**Authors:** Joseph Baruch Baluku, Resty Nanyonjo, Jolly Ayo, Jehu Eleazer Obwalatum, Jane Nakaweesi, Catherine Senyimba, Deus Lukoye, Joseph Lubwama, Jennifer Ward, Barbara Mukasa

**Affiliations:** 1 Mildmay Uganda, Wakiso, Uganda; 2 Makerere University Lung Institute, Kampala, Uganda; 3 Division of Global HIV and TB, US Centers for Disease Control and Prevention, Kampala, Uganda

**Keywords:** Case notification rate, Tuberculosis, Rural, Uganda, HIV, TB/HIV, Trends, Treatment success, TB

## Abstract

**Background:**

The End TB Strategy aims to reduce new tuberculosis (TB) cases by 90% and TB-related deaths by 95% between 2015 – 2035. We determined the trend of case notification rates (CNRs) and treatment outcomes of TB cases with and without HIV co-infection in rural Uganda to provide an interim evaluation of progress towards this global target in rural settings.

**Methods:**

We extracted retrospective programmatic data on notified TB cases and treatment outcomes from 2015 – 2019 for eight districts in rural Uganda from the District Health Information System 2. We estimated CNRs as the number of TB cases per 100,000 population. Treatment success rate (TSR) was calculated as the sum of TB cure and treatment completion for each year. Trends were estimated using the Mann–Kendall test.

**Results:**

A total of 11,804 TB cases, of which 5,811 (49.2%) were HIV co-infected, were notified. The overall TB CNR increased by 3.7-fold from 37.7 to 141.3 cases per 100,000 population in 2015 and 2019 respectively. The increment was observed among people with HIV (from 204.7 to 730.2 per 100,000, *p* = 0.028) and HIV-uninfected individuals (from 19.9 to 78.7 per 100,000, *p* = 0.028).

There was a decline in the TSR among HIV-negative TB cases from 82.1% in 2015 to 63.9% in 2019 (*p* = 0.086). Conversely, there was an increase in the TSR among HIV co-infected TB cases (from 69.9% to 81.9%, *p* = 0.807).

**Conclusion:**

The CNR increased among people with and without HIV while the TSR reduced among HIV-negative TB cases. There is need to refocus programs to address barriers to treatment success among HIV-negative TB cases.

**Supplementary Information:**

The online version contains supplementary material available at 10.1186/s12889-022-13111-1.

## Background

In 2019, tuberculosis (TB) was the leading cause of death from an infectious agent accounting for 1.2 million deaths among HIV-uninfected people and 208,000 deaths among HIV-infected individuals globally [1]. The End TB Strategy aims to reduce the TB incidence rate by 90% and reduce TB deaths by 95% between 2015 – 2035 [2]. An interim milestone for the year 2020 is to reduce TB incidence and deaths by 20% and 35% respectively. However, the global incidence of TB and TB deaths has fallen by only 8.8% and 14%, respectively, between 2015 and 2019 [1]. The global annual percentage change in the incidence rate (APCIR) was -1.1% between 2015 and 2017 against a target of -4%; for which only 2 of 21 Global Burden of Disease regions were on target [3].

Sub-Saharan Africa (SSA) accounts for a quarter of the global TB cases of which 32% are co-infected with HIV [4]. A recent analysis of TB case notification rates (CNR) between the year 2000 and 2018 from 58 countries found a 0.6% reduction in CNR in Africa that was partly attributed to the roll out and access to antiretroviral therapy (ART) among people with HIV [5]. Nevertheless, the region is not on course to realise the End TB Strategy goals. The East SSA region registered the least APCIR in SSA of -0.2% between 2015 – 2017 [3]. There is need to identify “hot spot” sub-regions where progress is slow. This would help in designing targeted interventions for specific sub-groups and areas.

Uganda is a TB/HIV high-burdened country where TB and TB/HIV co-infection show different spatial clustering patterns [6]. The decline in TB incidence in Uganda is slow due to the rise in the number of new TB cases among HIV-uninfected individuals, although HIV co-infected TB cases have declined between 2000 and 2018 [7]. Moreover, there is significant variation in treatment success rate (TSR) across the country among TB cases with and without HIV [8]. Rural settings in Uganda typically report a TSR of < 70% and are likely to lag behind their urban counterparts in achieving the End TB Strategy [8, 9]. In this study we determined the trend of the CNRs among individuals with and without HIV and treatment outcomes of TB cases with and without HIV co-infection in rural Uganda to provide an interim evaluation of progress towards the End TB Strategy in rural settings.

## Methods

### Study population and settings

This was a retrospective review of TB programmatic data from 8 districts of rural central Uganda (the “Mubende region”). The region is comprised of Mubende, Kiboga, Luweero, Nakaseke, Kassanda, Nakasongola, Mityana and Kyankwanzi districts of Uganda. As of 2019, the region had a population of 247,728 people with HIV and 2,328,472 HIV negative individuals (Supplementary table 1). In this region, Mildmay Uganda, a non-governmental organisation, with support from US President’s Emergency Plan for AIDS Relief (PEPFAR), through the US Centers for Disease Control and Prevention (CDC), provides technical support to public health facilities in the delivery of TB services. TB-related data are routinely generated at TB diagnostic and treatment units and entered in the unit TB registers. Subsequently, data are entered in the District Health Information System 2 (DHIS2), an information system used to document routinely collected health-related data across public health facilities [10]. In this analysis, we extracted data from DHIS2 for TB cases notified and treatment outcomes documented between 2015 – 2019, disaggregated by HIV status. Treatment outcomes were available for drug-susceptible TB cases only. The Ministry of Health in Uganda recommended patients with drug-susceptible TB to be initiated on a 6-months’ regimen with 2 months of rifampicin, isoniazid, ethambutol and pyrazinamide and a continuation phase of 4 months with rifampicin and isoniazid [11].

### Study measurements

Using a data abstraction form, the following variables were abstracted: number of TB cases notified by year, proportion of TB cases notified by HIV status, ART and cotrimoxazole use status among HIV-positive cases, district, age category (0 – 5, 5 – 14, > 15 years), level of health facility, sex, TB class (pulmonary bacteriologically diagnosed, pulmonary clinically diagnosed and extrapulmonary TB), TB treatment category (new and relapse, return after lost-to-follow-up, and failure), and drug resistance profile. In DHIS2, new and relapse cases were grouped together. TB treatment outcomes were cure, lost-to-follow-up, failure, death and transferred out as defined by WHO [12]. Treatment success was a sum of TB cure and treatment completion. We calculated the overall CNR as the proportion of TB cases notified in DHIS2 each year divided by the projected population of the individual districts (and the entire region as a sum of the individual districts population) and expressed per 100,000 population. The population size estimate of the individual districts for each year was obtained from the Uganda Bureau of Statistics estimates [13]. In estimating the number of people living with HIV in each district (Supplementary table 1), we used hybrid prevalence estimates for each district using health facility data in DHIS2 and survey data from the Uganda AIDS indicator survey [14]. As such, the prevalence of HIV for each district was estimated as: Kiboga (6.0% among men and 13.7% among women), Kyankwanzi (10.5% among men and 13.5% among women), Luwero (9.8% among men and 9.2% among women), Mityana (11.3% among men and 18.3% among women), Mubende (8.3% among men and 7.0% among women), Nakasongola (9.1% among men and 7.5% among women), and Nakaseke (7.3% among men and 7.4 among women) [14]. Kassanda district was part of Mubende district until 2018. Therefore, the HIV prevalence for Kassanda district was assumed to be the same as that for Mubende district. The trend of the prevalence of HIV has been level among rural residents in Uganda between 2011 – 2019 [15].

### Study outcomes

The study outcomes were the trend of the annual CNR and TB treatment outcomes for the region disaggregated by HIV status. Specifically, an overall CNR, the CNR among people with HIV and the CNR among HIV-uninfected individuals were estimated.

### Statistical analysis

Data were entered in Microsoft Excel® and analysed in Stata 16.0 (STATA, College Station, Texas, USA). We describe characteristics of TB cases by HIV status using proportions. We used the Mann–Kendall test to estimate the overall trend of CNRs and treatment outcomes over the period under study. We further analysed the trend of CNRs by HIV status, sex, district, type of TB case, TB class and resistance profile.

## Results

The period under study was 2015 – 2019. The analyses were performed between March and April 2021.

### Characteristics of TB cases notified in rural Uganda (2015 – 2019)

A total of 11,804 TB cases were notified of which 7,584 (64.2%) were male, 10,635 (90.1%) were aged ≥ 15 years and 5,811 (49.2%) were HIV co-infected TB cases. Of 5,811 HIV co-infected TB cases, 5,466 (93.7%) were on ART and 5,724 (98.5%) were on cotrimoxazole prophylaxis at the time of documenting the TB treatment outcome. Among the notified cases, 6,372 (54.0%) were pulmonary bacteriologically confirmed, 4,784 (40.5%) were pulmonary clinically diagnosed and 648 (5.5%) were extrapulmonary TB cases. A drug resistance profile was available for 1,269 (10.8%) cases, of which 1,188 (93.6%) had drug-susceptible TB. By TB category, 11,246 (95.3%) were new and relapse cases, 375 (3.2%) were return after lost-to-follow-up cases and 92 (0.8%) were treatment failure cases at initiation of treatment. The TB category was unknown for 91 (0.8%) cases. Table 1 shows the characteristics of the TB cases by HIV status.

**Table 1:** Characteristics of TB cases notified in eight districts of rural Uganda (2015—2019)

Characteristic	Total (*N* = 11,804)	HIV positive (*n* = 5811)	HIV negative (*n* = 5993)
**Year**			
2015	856 (7.3)	449 (7.7)	407 (6.8)
2016	1877 (15.9)	932 (16)	945 (15.8)
2017	2250 (19.1)	1046 (18)	1204 (20.1)
2018	3180 (26.9)	1575 (27.1)	1605 (26.8)
2019	3641 (30.8)	1809 (31.1)	1832 (30.6)
**District**			
Kassanda	834 (7.1)	404 (7)	430 (7.2)
Kiboga	1263 (10.7)	633 (10.9)	630 (10.5)
Kyankwanzi	1057 (9)	540 (9.3)	517 (8.6)
Luwero	2135 (18.1)	1112 (19.1)	1023 (17.1)
Mityana	2402 (20.3)	1290 (22.2)	1112 (18.6)
Mubende	2095 (17.7)	864 (14.9)	1231 (20.5)
Nakaseke	1143 (9.7)	572 (9.8)	571 (9.5)
Nakasongola	875 (7.4)	396 (6.8)	479 (8)
**Level of health facility**			
Regional-level referral hospital	1450 (12.3)	581 (10)	869 (14.5)
District-level hospital	2666 (22.6)	1368 (23.5)	1298 (21.7)
Health center IV	3045 (25.8)	1489 (25.6)	1556 (26.0)
Health center III	4204 (35.6)	2140 (36.8)	2064 (34.4)
Health center II	439 (3.7)	233 (4.0)	206 (3.4)
**Sex**			
Male	7584 (64.2)	3432 (59.1)	4152 (69.3)
Female	4220 (35.8)	2379 (40.9)	1841 (30.7)
**Drug resistance**^**a**^**, *n* = *81***			
RR TB	55 (67.9)	20 (64.5)	35 (70.0)
MDR TB	26 (32.1)	11 (35.5)	15 (30.0)

^a^data for susceptible TB cases were not disaggregated by HIV status from the data source (DHIS2). *RR TB* rifampicin resistant TB, *MDR TB* multidrug resistant TB

### Trend of overall TB CNRs among people with and without HIV in rural Uganda (2015 – 2019)

**Fig. 1:**
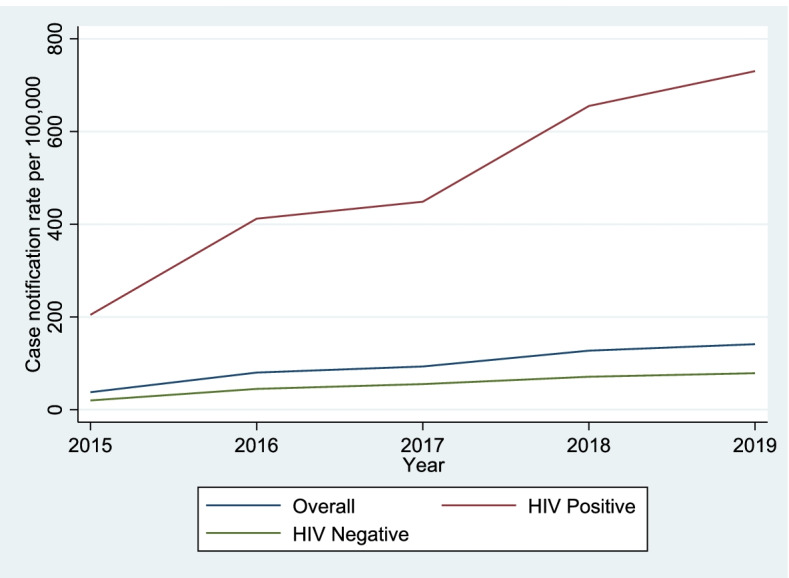
Trend of overall TB case notification rates (CNR) and CNR by HIV status of the population in eight districts of rural Uganda

The overall TB CNR increased by 3.7-fold from 37.7 to 141.3 cases per 100,000 population in 2015 and 2019 respectively. The increment was observed among people with HIV (from 204.7 to 730.2 per 100,000, *p* = 0.028) and HIV-uninfected individuals (from 19.9 to 78.7 per 100,000, *p* = 0.028). Figure 1 shows the trend of the overall CNR and notification rate by HIV status.

#### Trend of total TB CNRs in sub-groups

Table 2 summarises the total and sub-group CNRs among the cases. The CNRs increased in almost all sub-groups. We observed a significant positive trend in the CNRs of new and relapse cases, and pulmonary bacteriologically confirmed cases. Further, there was a significant positive trend in the CNR for both sexes (Additional file 1: Appendix 1) and most districts.

**Table 2:** Trend of overall TB CNRs among sub-groups in rural Uganda (2015—2019)

	**Total case notification rate (CNR)** **per 100,000 population**					*p*-value*
	**2015**	**2016**	**2017**	**2018**	**2019**	
**Overall**	37.7	80.1	93	127.4	141.3	0.028
**District**						
Kassanda	19.5	40.2	58.1	76.5	88.1	0.028
Kiboga	66.1	110.9	175.4	235.4	196.3	0.086
Kyankwanzi	17.5	58.1	92.8	102.1	144.8	0.028
Luwero	28.4	66.1	82.3	108.6	145.1	0.028
Mityana	72.3	132.4	124.0	202.8	160.3	0.221
Mubende	43.5	77.5	81.4	97.4	128.8	0.028
Nakaseke	27.2	113.2	99.7	130.1	153.6	0.086
Nakasongola	25.8	54.3	71.9	134.2	147.2	0.028
**Sex**						
Male	49.7	102.5	119.1	158.3	171.9	0.028
Female	25.3	56.7	65.7	94.7	109.0	0.028
**Tuberculosis (TB) class**						
PBC	21.6	50.7	53.3	61.4	72.8	0.024
PCD	11.8	22.4	35.2	60.8	63.0	0.060
EPTB	4.4	7.0	4.5	5.2	5.6	1.000
**TB treatment category**						
New and relapse	35.4	74.1	87.5	122.4	137.2	0.028
Return after lost-to-follow-up	1.5	3.8	2.8	3.9	3.3	0.462
Failure	0.7	0.9	0.9	0.6	0.7	1.000
History unknown	0.0	1.3	1.9	0.4	0.2	1.000
**Drug resistance**						
Susceptible TB	1.0	7.1	8.6	16.1	15.1	0.086
RR TB	0.3	0.2	0.6	0.5	0.7	0.221
MDR TB	0.0	0.1	0.2	0.4	0.3	0.086

*PBC* pulmonary bacteriologically confirmed, *PCD* pulmonary clinically diagnosed, *EPTB* extrapulmonary TB, *RR TB* rifampicin resistant TB, *MDR TB* multidrug resistant TB. *p-value from Mann–Kendall test

#### Trend of TB CNRs in sub-groups of people with and without HIV infection

The trend of the CNRs by HIV status could only be computed for districts, sex, and drug resistance status. There was a significant positive trend in the CNR of TB cases among people with HIV and HIV-uninfected people in both sexes and across most districts. Table 3 shows sub-group CNRs of TB cases by HIV status.

**Table 3:** Trend of TB CNRs in sub-groups among people with and without HIV in eight districts of rural Uganda

	**CNR among PWH** **per 100,000 population**					*p*-value*	**CNR among HIV-uninfected** **per 100,000 population**					*p*-value*
	**2015**	**2016**	**2017**	**2018**	**2019**		**2015**	**2016**	**2017**	**2018**	**2019**	
**Overall**	204.7	412.1	448.5	655.1	730.2	0.028	19.9	44.7	55.1	71.1	78.7	0.028
**District**												
Kassanda	98.9	234.2	362.8	480.7	601.4	0.028	12.9	24.0	32.8	42.9	45.4	0.028
Kiboga	359.4	588.9	845.7	1,254.0	990.9	0.086	34.4	59.1	103.1	125.7	110.7	0.086
Kyankwanzi	48.8	250.4	378.8	374.3	717.5	0.086	13.2	32.0	54.1	65.2	67.3	0.028
Luwero	185.5	337.7	405.1	620.8	810.2	0.028	11.9	37.5	48.4	54.8	75.3	0.028
Mityana	276.3	527.3	457.4	716.4	543.7	0.221	37.0	64.0	66.1	113.9	94.0	0.086
Mubende	255.16	423.9	391.6	564.6	675.4	0.086	25.9	48.8	55.6	58.7	83.5	0.028
Nakaseke	208.7	757.7	608.7	873.5	1,117.1	0.086	12.8	62.1	59.3	71.2	77.2	0.086
Nakasongola	187.2	263.1	383.0	743.6	779.8	0.028	11.1	35.3	43.6	78.8	89.7	0.028
**Sex**												
Male	259.2	523.2	553.9	806.7	860.4	0.028	28.8	60.6	75.7	93.7	103.3	0.028
Female	154.7	309.5	350.7	513.4	607.9	0.028	10.4	27.8	33.1	47.0	52.3	0.028
**Drug resistance**												
RR TB	0.5	1.3	0.4	3.3	2.8	0.462	0.3	0.4	0.6	0.2	0.4	1.00
MDR TB	0.0	0.9	0.4	3.3	1.2	0.221	0.0	0.0	0.1	0.2	0.2	0.221

### Treatment outcomes of TB cases in rural Uganda (2015 – 2019)

**Fig. 2:**
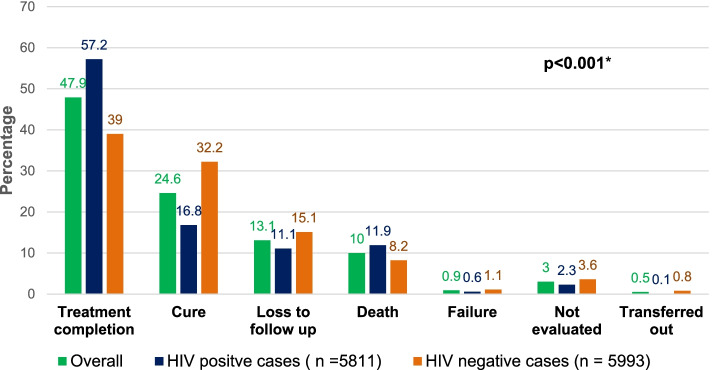
Treatment outcomes of TB cases in rural Uganda by HIV status (2015 – 2019). *p-value compares overall treatment outcomes among HIV positive and HIV negative cases

The overall TSR was 72.5% and was significantly lower among HIV-negative cases (71.2%, *N* = 5,993) compared to HIV positive cases (74%, *N* = 5,811) (*p* < 0.001). Treatment lost-to-follow-up rate (15.1% vs. 11.1%), failure rate (1.1% vs. 0.6%), and “not evaluated” (3.6% vs. 2.3%) were higher among HIV-negative TB cases compared to the HIV-positive TB cases while death was higher among HIV-positive cases compared to HIV-negative cases (11.9% vs. 8.2%), *p* < 0.001. Figure 2 summarises the treatment outcomes of TB cases by HIV status.

### Trend of TB treatment outcomes in rural Uganda (2015 – 2019)

**Fig. 3:**
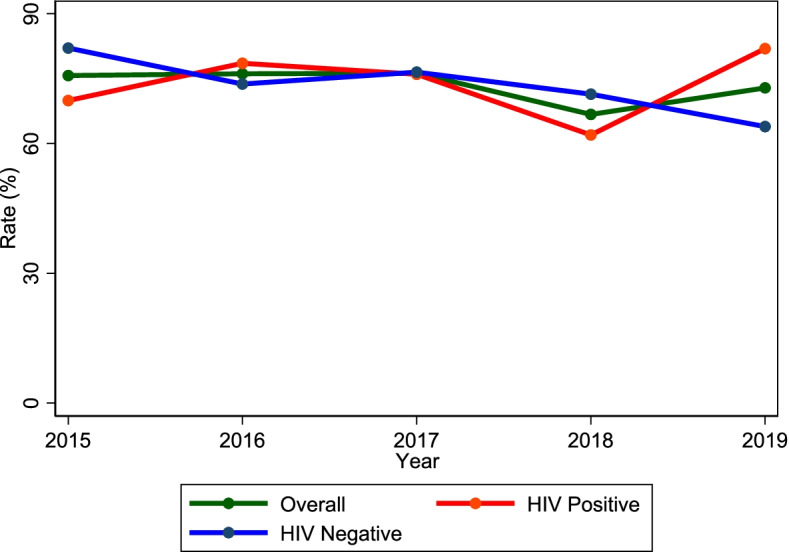
Trend of overall treatment success rate (TSR) and the TSR by HIV status (positive vs. negative) in rural Uganda

There was a decline in the TSR among HIV-negative TB cases from 82.1% in 2015 to 63.9% in 2019 (*p* = 0.086). Conversely, there was an increase in the TSR among HIV co-infected TB cases (from 69.9% to 81.9%, *p* = 0.807). The overall TSR was for the most part level (75.7% in 2015 and 72.9 in 2019, *p* = 0.807). Figure 3 shows the trend in the overall TSR and the TSR by HIV status. Table 4 shows the trends of the individual treatment outcomes (cure, treatment completion, lost-to-follow-up, death, failure, “transferred out” and “not evaluated”).

**Table 4:** Trends of TB treatment outcomes in rural Uganda (2015—2019)

	**Total (%)**					*p*-value*	**Outcome in HIV/TB cases** **(%)**					*p*-value*	**Outcome in HIV negative TB cases** **(%)**					*p*-value*
	**2015**	**2016**	**2017**	**2018**	**2019**		**2015**	**2016**	**2017**	**2018**	**2019**		**2015**	**2016**	**2017**	**2018**	**2019**	
**Treatment success**	75.7	76.1	76.3	66.7	72.9	0.807	69.9	78.5	76.0	62.0	81.9	0.807	82.1	73.8	76.5	71.4	63.9	0.086
**Cure**	24.9	27.2	28.7	23.2	22.0	0.462	17.8	20.0	19.0	17.5	13.2	0.221	32.7	34.4	37.1	28.8	30.7	0.807
**Treatment completion**	50.8	48.9	47.6	43.5	50.9	0.807	52.1	58.6	57.0	44.5	68.8	0.807	49.4	39.4	39.4	42.6	33.2	0.462
**Lost-to-follow-up**	11.4	10.4	12.7	15.0	13.5	0.221	18.0	5.5	10.6	18.0	6.7	0.807	4.2	15.3	14.5	12.1	20.3	0.462
**Death**	10.3	12.4	10.1	10.0	8.7	0.086	10.5	15.5	13.0	14.2	7.7	0.807	10.1	9.3	7.6	5.9	9.6	0.462
**Transferred out**	0.0	0.0	0.0	0.0	1.5	0.289	0.0	0.0	0.0	0.0	0.4	0.289	0.0	0.0	0.0	0.0	2.6	0.289
**Failure**	2.6	1.1	0.9	0.5	0.6	0.086	1.6	0.5	0.4	0.6	0.4	0.462	3.7	1.6	1.3	0.4	0.8	0.086
**Not evaluated**	0.0	0.0	0.0	7.7	2.8	0.267	0.0	0.0	0.0	5.2	2.8	0.267	0.0	0.0	0.0	10.2	2.8	0.267

^*^p-value from Mann–Kendall test

## Discussion

In this study we determined the trend of the TB CNRs among people with and without HIV and treatment outcomes of TB cases with and without HIV co-infection in eight districts of rural Uganda. We found that during 2015 -2019, the CNRs increased significantly among people with and without HIV. Additionally, the TSR reduced among HIV-negative but increased among HIV positive TB cases in this region.

The CNR of 141 per 100,000 population that we observed in 2019 is comparable to the national CNR (149 per 100,000 population) in the same year [1]. However, there are few reports from rural Uganda with which to compare the trend of the CNRs observed in our study. Similar to our finding, an increase in the number of notified cases was observed between 2015 – 2017 in a study that abstracted data from Kiboga, Mityana and Nakaseke district hospitals [16]. TB detection among HIV-infected and HIV-uninfected individuals could be increasing in this region. This might be attributed to an increase in access to TB diagnostic services following allocation of district-specific targets for TB notification by the national TB program. In this region, there has been improvement in implementing-partner support to TB program activities at public health facilities. Specifically, facilities have been supported to scale up TB screening, provide free chest X-ray vouchers to cover imaging costs and build the confidence of health workers, through mentorship activities, to clinically diagnose TB. This may have contributed to the increase in the CNRs in pulmonary bacteriologically confirmed TB and clinically diagnosed TB. However, the specific impact of these interventions on the trend of CNRs in rural Uganda needs to be evaluated further.

Uganda rolled out the Xpert MTB/RIF assay, a cartridge-based nucleic acid amplification test, for the diagnosis of pulmonary TB in 2012 and the use of the urine lipoarabinomannan (LAM) among ill people with HIV in 2017 [11]. However, the roll out of the Xpert MTB/RIF assay has seen very low utilisation rates among HIV-infected and HIV-uninfected individuals with suspected TB in rural settings [17]. Less than 20% of presumptive TB patients are referred for sputum evaluation with Xpert MTB/RIF assay [18]. Moreover, historically, the roll out of the Xpert MTB/RIF assay in Uganda has had no effect on the CNRs [19]. Further, we did not observe a significant increase in the CNRs of drug resistant TB for which the Xpert MTB/RIF assay is the commonest drug susceptibility test in Uganda. Additionally, the combination of the Xpert MTB/RIF assay and urine LAM in the diagnostic algorithm of TB results in a dismal (1 – 4%) increment in identified new cases [20, 21]. Therefore, it is unclear whether the increase in the CNR in our study is solely attributed to increased access to TB testing. The effect of the Xpert MTB/RIF assay and the urine LAM on the CNRs in rural settings needs to be evaluated by future studies.

The increase in pulmonary bacteriologically confirmed and new/relapse cases observed in the study is concerning as it suggests an increased risk of TB transmission in rural settings. Several factors in rural settings could facilitate TB transmission. Rural settings in Uganda are experiencing population growth, urbanization and lifestyle changes that could increase the risk for TB infection [13, 22]. Poverty levels, a key risk factor for TB, have also been increasing in Uganda over the period under study. Poverty levels in central Uganda increased from 4.7% in 2012 to 12.7% in 2017 [23]. Further, cigarette smoking and alcohol use, which are other risk factors for TB, positively correlate with poverty levels in rural Uganda [24]. Also, the prevalence of HIV has stagnated over the last decade in rural Uganda where men, the most-at-risk gender for TB, have a higher incidence of HIV infection than urban men [15]. From our study, the frequency of TB/HIV co-infection was stable across the period of study (between 49%—52%). More studies are needed to ascertain whether the increase in the CNRs is due to TB transmission or detection in rural settings. There is, also, a need to increase uptake of TB preventive therapies and intensify case finding in rural areas. An increase in the TB CNR in rural settings has also been reported in Ethiopia which was attributed to increased access and utilisation of TB services particularly in the older populations [25]. In Uganda, the incidence of TB has dropped by only -1% between 2015 – 2019 [1]. This reduction is small and likely to stem from a reduction of TB incidence in urban settings [26–28]. WHO has recently redesignated Uganda as a TB high-burdened country [29]. The contribution of rural settings to the high burden of TB in Uganda needs to be addressed.

From our findings it remains unclear why the TSR decreased from 82 to 64% among HIV-uninfected individuals over the study period. We observed a decrease in the rate of cure and treatment completion among HIV-negative cases and a higher rate of TB lost-to-follow-up and failure. Moreover, more HIV-negative cases were either transferred out or not evaluated. It is likely that a combination of these factors affected TSR among HIV-negative TB cases. The decline in the TSR among HIV negative cases in the face of an increasing CNR in HIV negative individuals is worrying. It implies that interventions to increase CNRs without a concurrent focus on ensuring treatment completion will result in higher rates of treatment attrition and failure as observed in this study. Moreover, cases that are lost to follow up or fail treatment propagate community transmission of TB and drug resistant TB. The Uganda national TB program aims to have a < 5% rate of lost-to-follow-up [30]. Therefore, the overall lost-to-follow-up rate among HIV negative cases in our study is thrice the target. This is alarming and deserves further evaluation. People with HIV are usually more integrated in the health care system and any disengagement with the system prompts tracing of the person by both TB and HIV care teams. This could explain the higher TSR in HIV-positive TB cases. Additionally, HIV programming in Uganda receives considerable funding from PEPFAR which could explain why the TSR and lost-to-follow-up rate among TB/HIV cases improved from 69.9% and 18.0% in 2015 to 81.9% and 6.7%, in 2019 respectively. Organisations implementing HIV care activities in districts sometimes run siloed activities that focus on achieving treatment success in HIV/TB co-infected cases, although they report to the ministry of health through the health information management systems [31]. This can inadvertently affect TSR in HIV-negative cases. Programs need to identify and address these disparities in treatment success in HIV-negative and positive TB cases in rural settings. In Uganda, creating incentives for TB focal persons at health facilities and improving the implementation of community-based directly observed therapy short course strategy might improve the TSR in rural areas [32].

Similar to our findings, a decline in the TSR was observed between 2015 – 2017 (from 73.4% to 64.4%) in a study that included data from Kiboga, Mityana and Nakaseke district hospitals, although data were not reported by HIV status [16]. Likewise, the majority with an unfavourable outcome in that study were mostly lost-to-follow up. However, unlike our findings that show a relatively high overall TSR (74%) among HIV positive cases, Musaazi and colleagues found the TSR to be 67% in a study that included Kiboga and Kyankwanzi districts [9]. However, almost all (92%) of their cases were treated with a less efficacious TB regimen consisting of 2 months of rifampicin, isoniazid, ethambutol and pyrazinamide and a continuation phase of 6 months with ethambutol and isoniazid [33].

Our study has limitations. We could not assess predictors of treatment success to explain why the TSR among HIV-negative cases was on a decline. Patient-level data were unavailable to us to conduct this analysis. The use of secondary data could introduce documentation bias. Treatment outcomes and notification could have been preferentially documented among TB cases with HIV co-infection since they are perceived to be at risk of TB disease and mortality. Lastly, the trends in the sub-group analyses should be interpreted with caution because of the small number of cases in these categories.

## Conclusion

The CNR increased among people with and without HIV in rural Uganda between 2015-2019. The TSR reduced among HIV-negative TB cases but increased among HIV negative cases. The results highlight a gap in TB prevention services in rural settings. Refocusing programs to address barriers to treatment success among HIV-negative TB cases is important for programs to achieve the goals of the End TB strategy.

## Supplementary Information

**Additional file 1: Appendix 1. Trends of TB treatment outcomes ***among men and women *in rural Uganda*disaggregated by HIV status* (2015 - 2019). **Supplementary table 1. **Population estimates for people with and without HIVin eight districts of rural Uganda.

## Data Availability

Datasets used in this analysis are available from the corresponding author on reasonable request.

## Abbreviations

CNR: Case notification rate
HIV: Human immunodeficiency virus
TB: Tuberculosis
TSR: Treatment success rate
SSA: Sub-Saharan Africa
ART: Antiretroviral therapy
CDC: Centers for Disease Control and Prevention
DHIS2: District health information system 2
CI: Confidence interval
LAM: Lipoarabinomannan
WHO: World health organisation
