# Supplementary material for: Trends of notification rates and treatment outcomes of tuberculosis cases with and without HIV co-infection in eight rural districts of Uganda (2015 – 2019)
Source: BMC Public Health. 2022 Apr 5;22:651. doi: 10.1186/s12889-022-13111-1 (PMC8981742; doi:10.1186/s12889-022-13111-1)
Supplement: Supplementary file 1 — Additional file 1: Appendix 1. Trends of TB treatment outcomes among men and women in rural Ugandadisaggregated by HIV status (2015 - 2019). Supplementary table 1. Population estimates for people with and without HIVin eight districts of rural Uganda. [file 12889_2022_13111_MOESM1_ESM.docx]

**Appendix 1**

**Trend of TB case notification rates among people without HIV**

**Trend of TB case notification rates among people with HIV**

**Trends of TB treatment outcomes *among men and women* in rural Uganda *disaggregated by HIV status* (2015 - 2019)**

**Supplementary table 1: Population estimates for people with and without HIV in eight districts of rural Uganda**

|  | **2015** | | **2016** | | **2017** | | **2018** | | 2019 | |
| --- | --- | --- | --- | --- | --- | --- | --- | --- | --- | --- |
| **District** | **Positive** | **Negative** | **Positive** | **Negative** | **Positive** | **Negative** | **Positive** | **Negative** | **Positive** | **Negative** |
| Kassanda | 21223 | 255,577 | 21774 | 262,126 | 22325 | 268,675 | 22884 | 275,316 | 23444 | 281,956 |
| Kiboga | 14747 | 136,453 | 15112 | 139,988 | 15490 | 143,610 | 15869 | 147,231 | 16248 | 150,852 |
| Kyankwanzi | 26646 | 196,554 | 27951 | 206,249 | 29304 | 216,296 | 30726 | 226,874 | 32196 | 237,804 |
| Luwero | 44215 | 421,285 | 45300 | 431,600 | 46405 | 442,095 | 47519 | 452,681 | 48633 | 463,267 |
| Mityana | 49220 | 284,080 | 50069 | 289,131 | 50936 | 294,264 | 51784 | 299,316 | 52603 | 304,197 |
| Mubende | 32932 | 397,268 | 34682 | 418,318 | 36516 | 440,384 | 38435 | 463,465 | 40422 | 487,378 |
| Nakaseke | 14855 | 187,345 | 15310 | 193,090 | 15772 | 198,928 | 16256 | 205,044 | 16740 | 211,160 |
| Nakasongola | 15490 | 170,610 | 15962 | 175,739 | 16449 | 181,051 | 16945 | 186,455 | 17442 | 191,859 |
| **Regional totals** | **219,328** | **2,049,171** | **226,160** | **2,116,242** | **233,197** | **2,185,303** | **240,418** | **2,256,382** | **247,728** | **2,328,472** |

Data sources:

1. Uganda Bureau of Statistics. Population Projections By District, 2015 to 2021 – Uganda Bureau of Statistics [Internet]. [cited 2021 May 18]. Available from: <https://www.ubos.org/population-projections-by-district-2015-to-2021/>
2. Ouma J, Jeffery C, Valadez JJ, Wanyenze RK, Todd J, Levin J. Combining national survey with facility-based HIV testing data to obtain more accurate estimate of HIV prevalence in districts in Uganda. BMC Public Health. 2020 Mar 23;20(1):379.
